# Rosai–Dorfman disease of the central nervous system: A clinical, radiological, and prognostic study of 12 cases

**DOI:** 10.3389/fonc.2022.1013419

**Published:** 2022-11-03

**Authors:** Xingshu Zhang, Wen Yin, Youwei Guo, Yi He, Zhipeng Jiang, Yuzhe Li, Bo Xie, Seng Zhang, Xingjun Jiang, Qing Liu, Jian Yuan

**Affiliations:** ^1^ Department of Neurosurgery, National Clinical Research Center for Geriatric Disorders, Xiangya Hospital, Central South University, Changsha, China; ^2^ Institute of Skull Base Surgery and Neuro-Oncology at Hunan, Changsha, China

**Keywords:** rosai-dorfman disease, central nervous system, magnetic resonance imaging, meningiomas, therapy

## Abstract

**Background:**

Rosai–Dorfman disease (RDD) is a rare benign non-Langerhans cell histiocytic proliferative disease. RDD with central nervous system (CNS) involvement (CNS-RDD) is extremely rare. Its etiology is unclear, and there are no consensus recommendations for its treatment. More studies are needed to elucidate the clinical and radiological manifestations and prognosis of CNS-RDD.

**Methods:**

From January 2012 to June 2022, 12 patients with CNS-RDD (intracranial or spinal) were retrospectively evaluated, including collecting clinical data, imaging data, and pathological findings; summarizing imaging characteristics; and conducting follow-up studies on CND-RDD patient treatment and prognosis.

**Results:**

Twelve CNS-RDD patients (nine male and three female patients, aged 12–67 years) were enrolled in this study. Nine patients represented convex and/or skull base RDD (eight with edema, six with lobulation and/or pseudopodium sign, four with multiple intracranial lesions), two patients had parenchymal RDD, and one patient had spinal cord subdural lesions. Symptoms of patients would vary according to the locations of the lesion, including but not limited to headaches, dizziness, seizures, cranial nerve dysfunction, and visual impairment. The immunohistochemistry of RDD showed positive expression of S100 and CD68 but not CD1a. Total resection (*n* = 7), subtotal resection (*n* = 3), partial resection (*n* = 1), and stereotaxic biopsy (*n* = 1) were achieved, respectively. A combination of chemotherapy plus steroid therapy was performed on two patients (relapsing case and residual lesion) and showed a remarkable effect.

**Conclusion:**

CNS-RDD, as a rare disease, presents a significant diagnostic challenge for clinicians. Solitary CNS-RDD are easily misdiagnosed as meningioma. However, when the MRI imaging of the disease represents dura-based masses with significant edema, homogeneous enhancement, lobulation, and/or pseudopodium sign, we should consider it might be the CNS-RDD. Surgery is an important and effective therapy for CNS-RDD. Steroids and chemotherapy are safe and effective for the postoperative treatment of relapsing cases or residual lesions.

## Introduction

Rosai–Dorfman disease (RDD), firstly recognized in 1969 by Rosai and Dorfman, is a rare, benign idiopathic histiocytic proliferation disease of uncertain etiology (1). Classically, RDD presents as massive bilateral cervical lymphadenopathy, but 43% of patients have extranodal involvement (2). RDD can be accompanied by systemic symptoms such as malaise, fever, weight loss, leukocytosis, and anemia. The prevalence of RDD is approximately one in 200,000. Only fewer than 5% of RDD cases involve the central nervous system (CNS), of which about 75% present as intracranial lesions and 25% occur in the spine.

For intracranial RDD, the most common symptoms include headache, epilepsy, and neurological deficits. Spinal RDD can cause compression to spinal segments and lead to sensory and/or motor dysfunction in the limbs (3, 4). The most common sites for intracranial RDD are the cerebral convexity and parasagittal region, parasellar and cavernous sinus region, and petroclival region (2, 5). Radiologically, the magnetic resonance imaging (MRI) manifestations of intracranial RDD are similar to meningioma with homogeneous enhancement and dural tail sign (6). Histologically, positive staining of S-100 and CD-68 but not CD1a is the specific immunohistochemical manifestation of RDD (7).

RDD with CNS involvement (CNS-RDD) has atypical clinical manifestations and imaging characteristics, making its accurate preoperative diagnosis challenging (2, 8). Moreover, few studies focused on the clinical and radiological presentations and prognosis of CNS-RDD, which still lacks a standardized treatment regimen. Thus, in this study, we summarized the clinical manifestations and imaging characteristics of 12 cases from a single center and reviewed the literature to promote awareness, diagnosis, and treatment of CNS-RDD.

## Material and methods

This study was approved by the ethics committee of Xiangya Hospital of Central South University, and informed consent was obtained from each patient. Twelve patients pathologically diagnosed as CNS-RDD at Xiangya Hospital between January 2012 and June 2022 were enrolled in this study. Their clinical characteristics of sex, age, CNS involvement sites, clinical presentations, and specific treatments were collected for further analysis. All these patients underwent conventional brain MRI enhancement scans with T1-weighted, T2-weighted, and T1-weighted enhancement images. The extent of tumor resection and recurrence was assessed by reviewing the MRI images. Postoperative patients were followed up regularly by telephone.

## Results

### Clinical features of patients enrolled

We summarized the detailed clinical features in **Table 1**. There were nine male and three female patients (a male-to-female ratio of 3:1) diagnosed with CNS-RDD in our institution. The average age of these patients was 41 years old, ranging from 12 to 67 years old. The most common symptoms among these patients were headaches, dizziness, seizures, cranial nerve dysfunction, and visual impairment. The most common locations were the cerebral convexity (including the frontal, parietal, temporal, and occipital lobes), followed by the petroclival region and sellar and parasellar regions. Other areas included the brain stem, thoracic spinal cord, and ventricle. Thus, the cerebral convexity and skull base were the predilection sites for CNS-RDD, which resembled the meningioma.

**Table 1:** Clinical profile of 12 patients with RDD at presentation.

Cases	Age (years)/sex	Tumor location(s)	Presentation	Within/external to nodes	Treatment	MRI features	Outcomes and follow-up
1	14/F	Left frontal lobe and lateral ventricle	Headache with nausea and vomiting	Purely extranodal	Biopsy, other treatment unknown	Homogeneous enhancement, peripheral edema	94 months, disease stable
2	57/F	Spinal cord, T10–T12	Numbness and weakness in the lower limbs	Both nodal and extranodal	Total resection	Homogeneous enhancement	86 months, complete remission
3	67/M	Right frontal and temporal region	Seizure	Purely extranodal	Total resection	Homogeneous enhancement, peripheral edema, lobulation, multiple lesions	16 months, seizure (controlled by drugs), complete remission
4	49/M	Left frontal and clivus region	Seizure	Purely extranodal	Total resection	Dura-based, homogeneous enhancement, peripheral edema, multiple lesions	55 months, complete remission
5	46/M	Left occipital lobe and transverse sinus	Headache	Purely extranodal	Total resection	Homogeneous enhancement, dura-based, lobulation/”pseudopodium” sign	49 months, complete remission
6	49/M	Left parietal region	Numbness of right upper and lower limbs	Purely extranodal	Total resection + chemotherapy + steroid therapy	Homogeneous enhancement, dura-based, peripheral edema, “pseudopodium” sign	37 months, recurrence (6 months), cytarabine + lenalidomide + dexamethasone, then complete remission
7	25/M	Right frontal and temporal region	Diplopia and headache	Purely extranodal	Total resection	Homogeneous enhancement, peripheral edema, dura-based	61 months, complete remission
8	49/M	Brain stem and bilateral cerebellum	Dizziness and numbness in the left limb	Both nodal and extranodal	Subtotal resection + steroid therapy	Homogeneous enhancement, peripheral edema, multiple lesions	41 months, prednisone progressed (paralysis of limbs)
9	12/M	Right frontal and temporal region, parasellar region	Intermittent dizziness	Purely extranodal	Subtotal resection + chemotherapy + steroid therapy	Homogeneous enhancement, dura-based, peripheral edema, lobulation	49 months, methotrexate + mercaptopurine + prednisone + dasatinib, disease stable
10	40/F	Sellar and parasellar regions	Headache, dizziness, ptosis, and limited abduction (left)	Purely extranodal	Subtotal resection	Homogeneous enhancement, dura-based	61 months, disease stable
11	51/M	Left petroclival region, cavernous sinus, and right parasellar region	Dizziness and right limb weakness	Purely extranodal	Partial resection	Multiple lesions, homogeneous enhancement, peripheral edema, dura-based	51 months, death after surgery, because of kidney failure and pulmonary infection
12	35/M	Right anterior and middle cranial fossa	Headache and vision loss	Purely extranodal	Total resection	Homogeneous enhancement, dura-based, “pseudopodium” sign	3 months, complete remission

### Radiological and pathological characteristics of CNS-RDD

**Figure 1:**
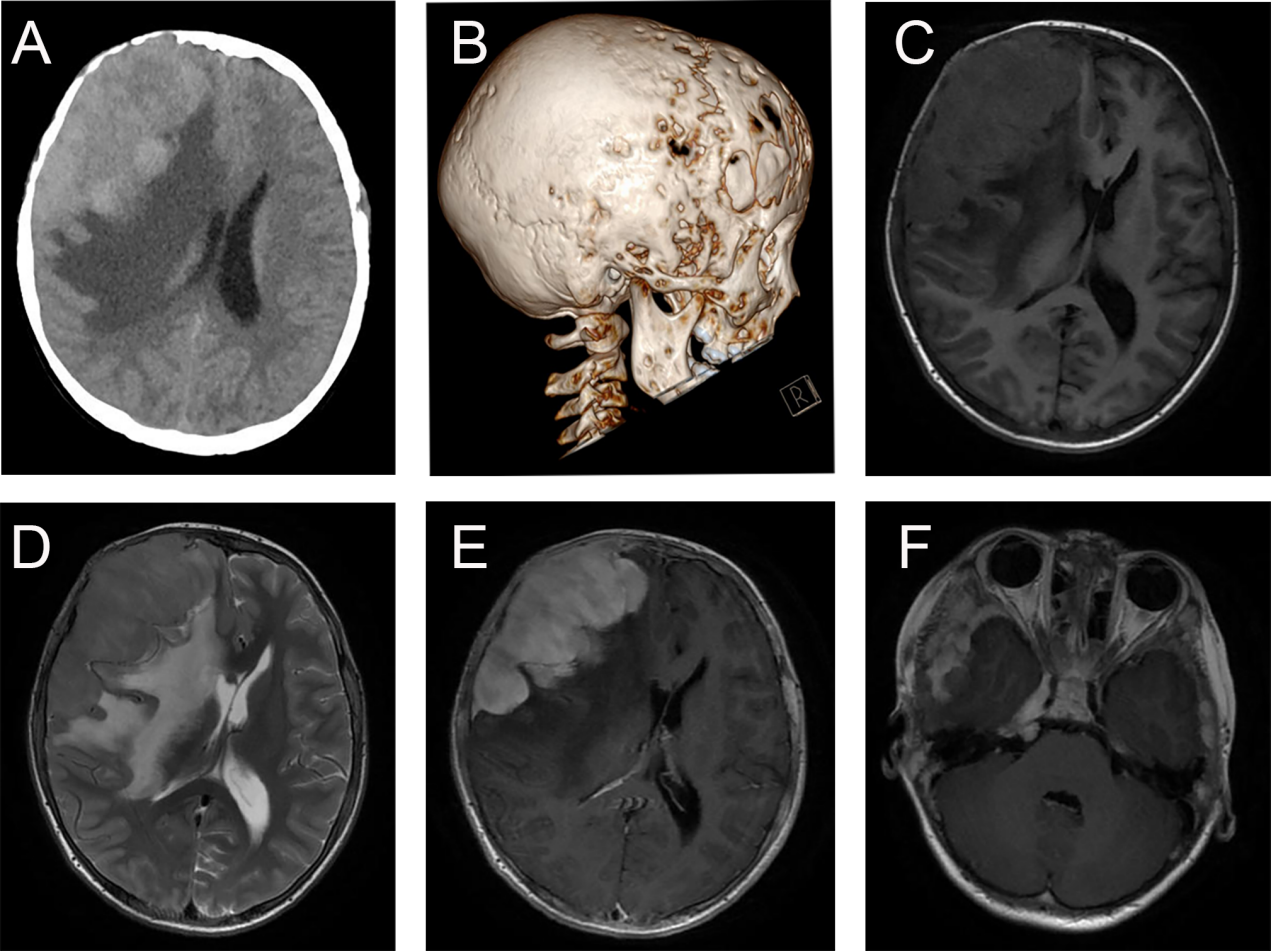
Typical imaging features of CNS-RDD (case 9). **(A, B)** CT images of axial cut and three-dimensional reconstruction showed destruction of the adjacent right frontal bone and a homogenous hyperattenuating mass with peripheral edema. MRI images with axial cuts show a homogeneous hypo- to the isointense mass of the right frontotemporal lobe on T1WI **(C)** and T2WI **(D)**, with significant homogeneous enhancement and dural attachment **(E)**. **(F)** Axial postcontrast T1WI showed CNS-RDD could also involve the right parasellar region.

**Figure 2:**
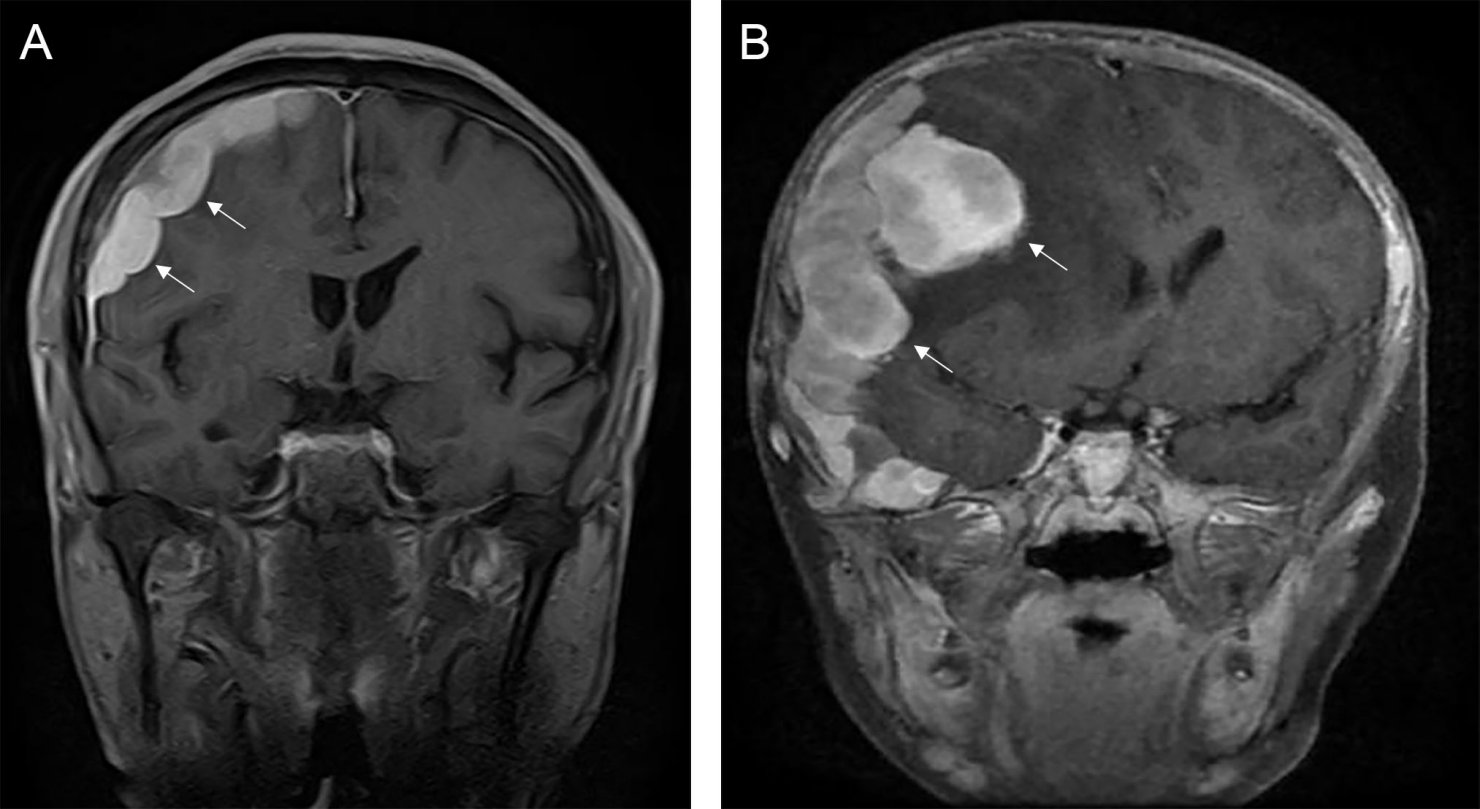
The lobulation sign of CNS-RDD. **(A, B)** MRI images showed the margin of the lesion is lobulated (arrow) on coronal contrast-enhanced images.

**Figure 3:**
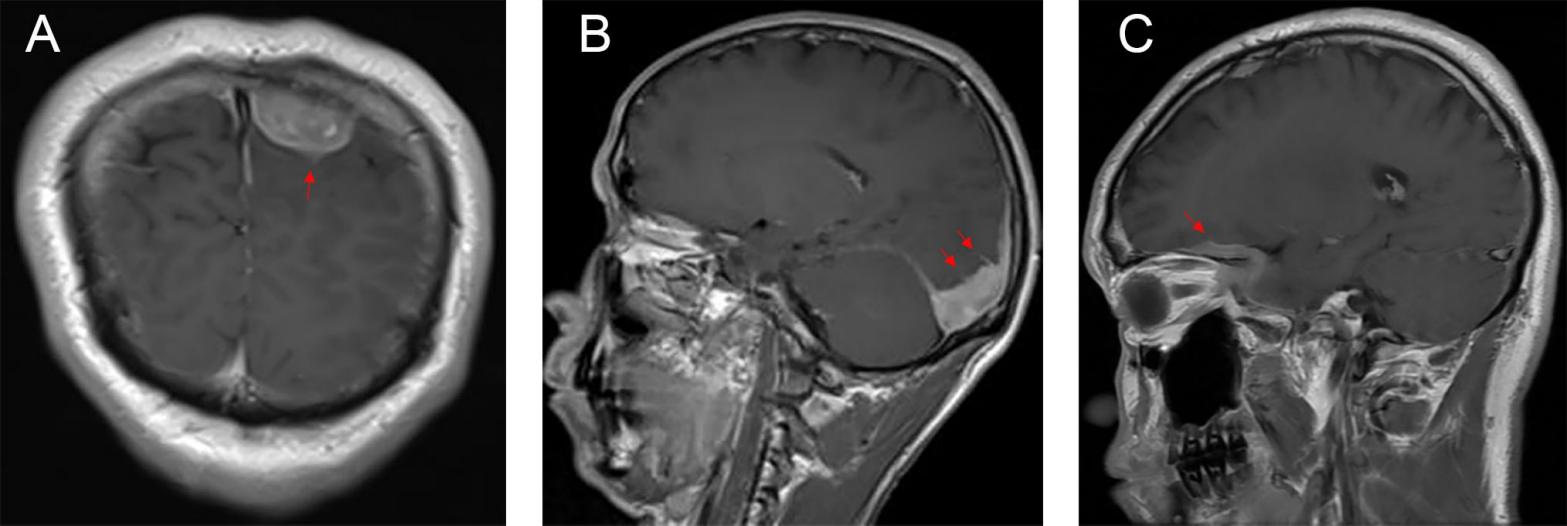
Postcontrast MRI images show that the irregularly thickened meningeal had a “pseudopodium” extending to the brain parenchyma (red arrow). **(A)** Coronal postcontrast T1WI. **(B, C)** Sagittal postcontrast T1WI.

**Figure 4:**
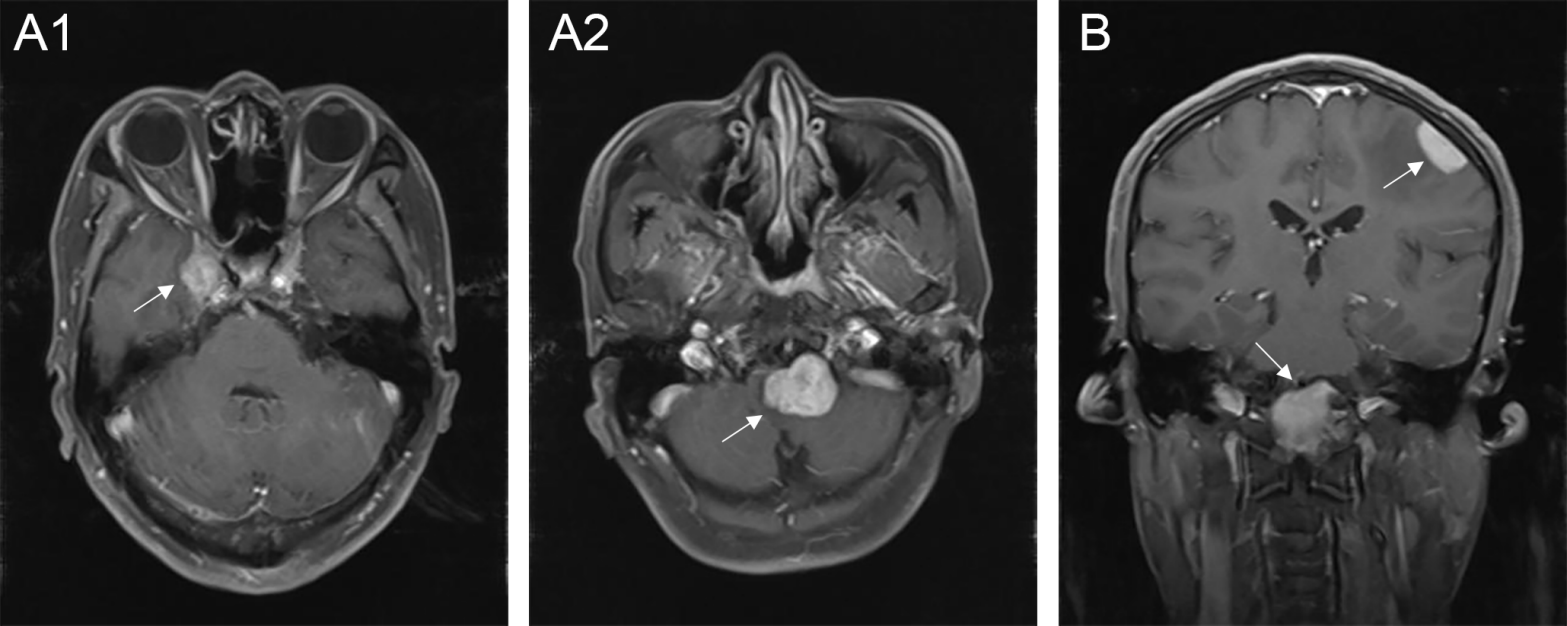
Multiple lesions of CNS-RDD in different intracranial parts. **(A1, A2)** Axial postcontrast T1WI showed enhanced multiple lesions (arrow) in the right parasellar and the clivus of one patient. **(B)** Coronal postcontrast T1WI showed enhanced multiple lesions (arrow) in the left convex and the clivus.

The typical computed tomography (CT) feature of the convex RDD is homogenous or slightly hyperattenuating mass with peripheral edema and with thickening or destruction of adjacent bone (**Figures 1A, B**). On MRI, most of the convex RDD were homogeneously hypo- to isointense on T1- and T2-weighted images (T1WI and T2WI), with significant homogeneous enhancement and dural attachment following contrast administration (**Figures 1C–F**). Preoperatively, all of the convex and/or skull base RDD patients (*n* = 9) were misdiagnosed with meningioma based on symptoms and radiological results. We found that lobulation and/or pseudopodium sign (5/9) are important radiological characteristics of CNS-RDD. On contrast-enhanced T1WI, the margin of the lesion is lobulated (**Figures 2A, B**), and/or the irregularly thickened meningeal had a “pseudopodium” extending to the brain parenchyma (**Figures 3A–C**). Four convex and/or skull base RDD (4/9) had multiple lesions in different intracranial parts, among which two cases showed lesions in both the supratentorial and infratentorial regions (**Figures 4A, B**).

**Figure 5:**
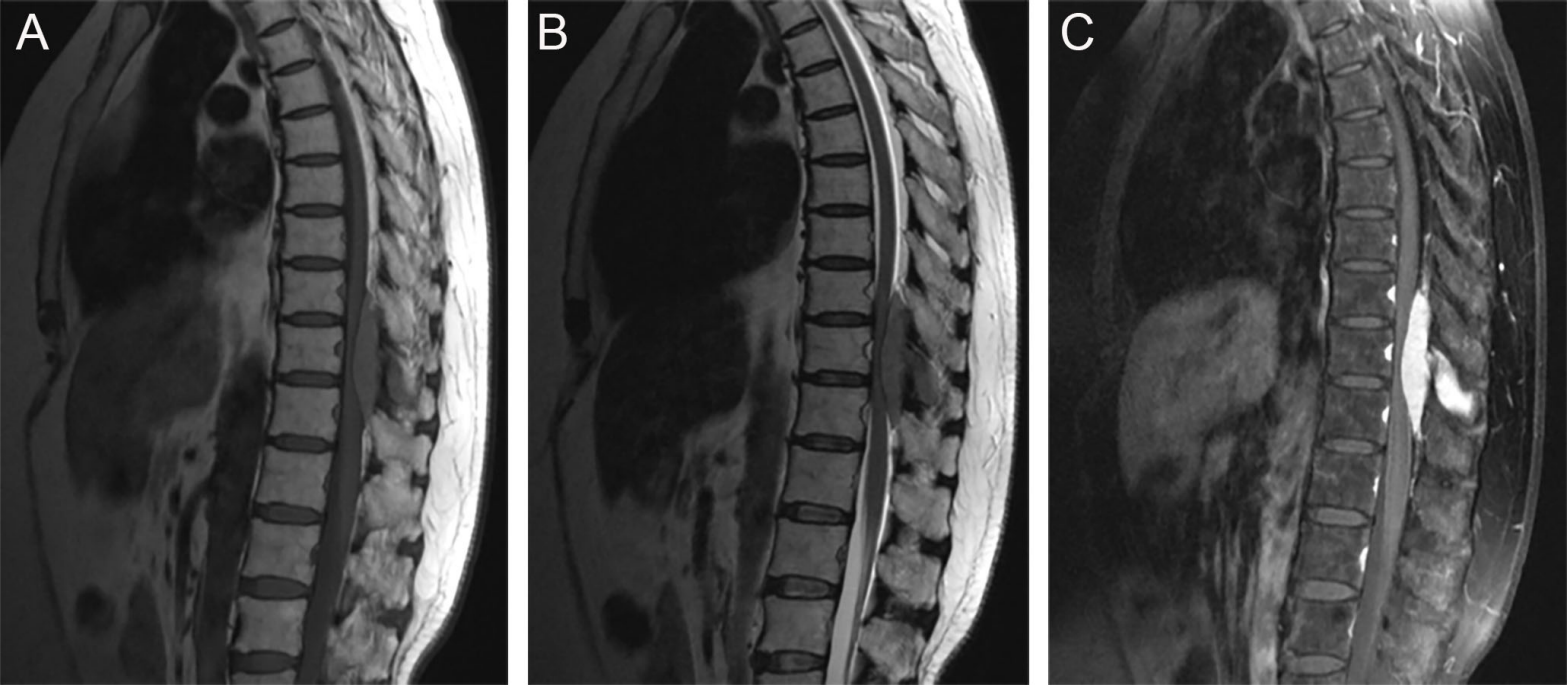
Sagittal MRI images showed a well-circumscribed extramedullary lesion with the destruction of adjacent bone, ranging from T10 to T12. **(A)** T1WI; **(B)** T2WI; **(C)** postcontrast T1WI.

There were three rare and noteworthy cases of CNS-RDD in our study: one case of intraventricular RDD (case 1), one case of brain stem RDD (case 8), and one case with thoracic spinal involvement (case 2). The MRI of case 1 displayed a lesion with the presence of an irregular shape, peripheral edema, ambiguous brain-lesion border, and moderate enhancement (**Supplementary Figures S1A–C**). The MRI of case 8 presented multiple lesions, moderate peripheral edema, and homogenously enhanced mass located in the pons (**Supplementary Figures S1D–F**). For the lesion located in the spine, MRI showed a homogenously enhanced and well-circumscribed extramedullary lesion with the destruction of adjacent bone, ranging from T10 to T12 (**Figures 5A–C**).

**Figure 6:**
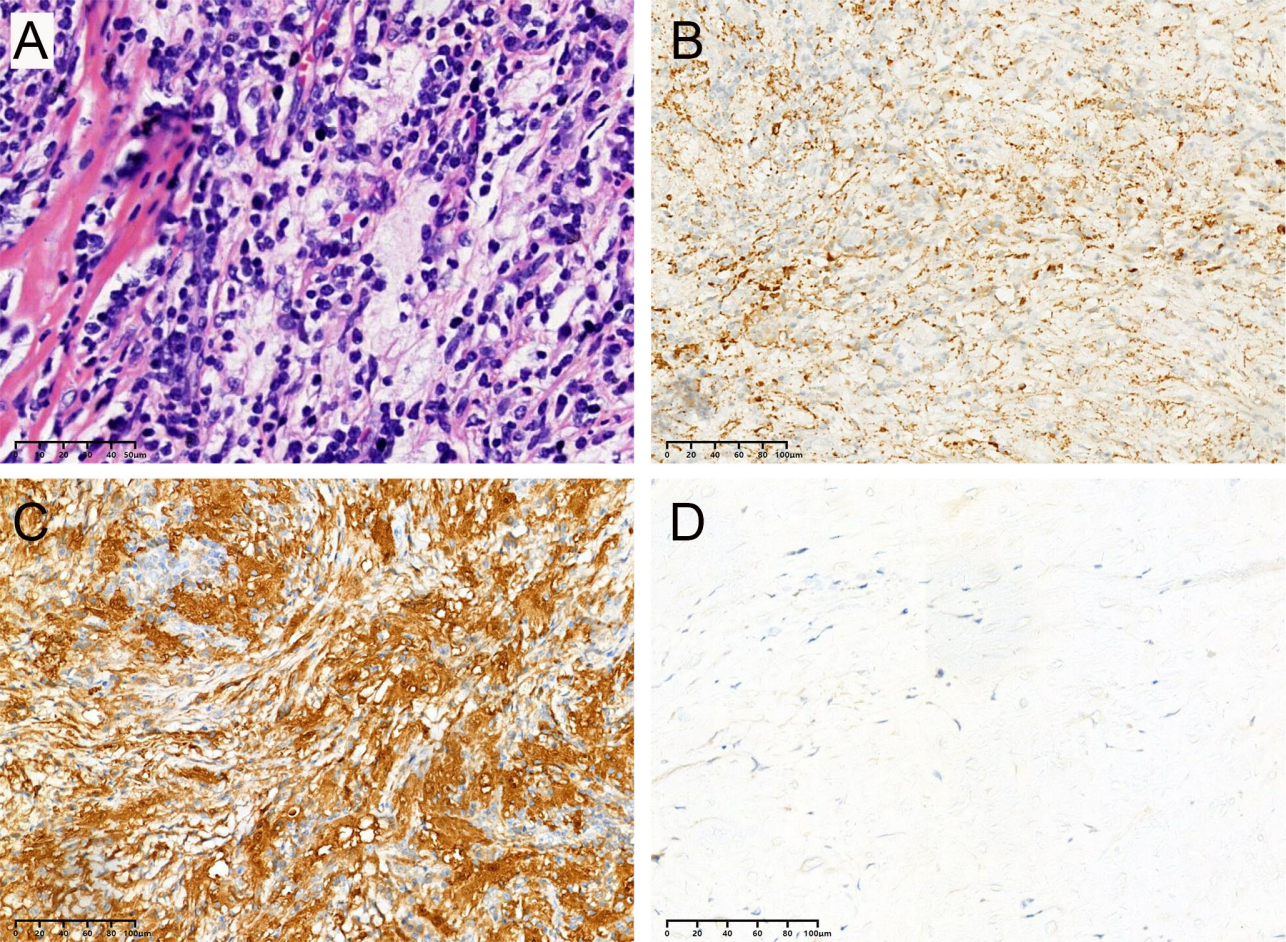
All cases in this series had similar morphologic features, and representative images are shown. **(A)** Biopsies contained an abnormal, vaguely nodular, polymorphous lymphohistiocytic infiltrate composed of abundant, loosely cohesive large histiocytes admixed with numerous small mature lymphocytes and plasma cells (hematoxylin and eosin). The immunohistochemical results showed positive expression of CD68 **(B)** and S-100 **(C)** protein but not CD1a **(D)**.

Most lesions were rich in blood supply, tough in texture, and closely adhered to the dura, brain parenchyma, and blood vessels. Histopathological examination showed histiocytes mixed with lymphocytes and plasma cells (**Figure 6A**). The immunohistochemical results proved positive expression of CD68 and S-100 protein, but not CD1a (**Figures 6B–D**).

### Surgery resection is an effective treatment for CNS-RDD

For being misdiagnosed with tumor, all (*n* = 12) of the patients received surgery as the first treatment. One patient received a stereotaxic biopsy (case 1) because the lesion was irregularly shaped and widespread around the lateral ventricle. Among the remaining patients, seven patients achieved total resection, three patients received subtotal resection, and one patient underwent partial resection. Case 4 with frontal and foramen magnum lesions achieved total resection at one-stage surgery. For case 10, as the lesion progressed 3 months after partial resection at a local hospital, we performed the second operation to remove the progressive sellar RDD. Among the seven patients who achieved total resection, the majority (6/7) experienced no recurrence during the follow-up, indicating that the total resection of CNS-RDD had a profound curative effect.

### Combination of chemotherapy plus steroid therapy showed promising therapeutic efficacy on recurrent and/or residual CNS-RDD

A combination of chemotherapy plus steroid therapy was performed on two patients (cases 6 and 9), and one patient (case 8) received steroid therapy. Case 6, complaining of left limb numbness, was admitted to the hospital with a diagnosis of parietal region meningioma. The lesion was then totally resected but pathologically diagnosed as RDD. Unfortunately, the lesion relapsed in the clivus region half a year after the operation. So, the patient was administered intravenous cytarabine (0.5 g/m^2^) every 12 h for 3 days, one cycle every 5 weeks for a total of five cycles (6 months). The patient then received oral lenalidomide (25 mg on days 1–21) and dexamethasone (40 mg on days 1, 8, 15, and 22) every 28 days for 6 months. The lesions disappeared completely, and no recurrence was found in regular follow-ups (**Supplementary Figures S2A, B**).

Case 9 presented with intermittent dizziness, and MRI showed homogenously enhanced lesions in the right frontotemporal and parasellar regions. Through the operation, the frontotemporal lesion (but not the parasellar one) was completely resected. The patient was then recommended to take monthly vinblastine 1.5 mg/m^2^, daily 6-mercaptopurine 50 mg/m^2^, daily prednisone 40 mg/m^2^, and weekly methotrexate 20 mg/m^2^ for four to six cycles. Although the patient had finished only one cycle of treatment before withdrawal, the disease remained stable at the latest follow-up.

Case 8 relapsed with paralysis of limbs a year after surgery and then received oral steroid therapy of 40 mg prednisolone daily. Unfortunately, the disease progressed during the subsequent follow-up.

### Detailed outcomes of patients followed up

All patients were followed-up after discharge. The mean follow-up time is 50 months (excerpt one death), ranging from 3 to 94 months. One patient (case 11) died 1 month after surgery because of kidney failure and pulmonary infection. Case 8 relapsed with limb paralysis 12 months after surgery, which still progressed after further steroid therapy, with complete paralysis of the four limbs at the latest follow-up. The postoperative lesion-related epilepsy occurred in one patient (case 3), which was controlled by taking an anti-seizure drug regularly. The rest of the patients were stable or in complete remission at the latest follow-up.

## Discussion

RDD, as a benign lymphohistioproliferative disease, is rarely involved in the CNS (9). It was a challenge to make an accurate diagnosis before surgery because the CNS-RDD lacked typical clinical manifestations and specific imaging features. Moreover, there is no consensus or guidelines on the treatment of CNS-RDD, and the clinical treatment mainly depends on the experience of the clinician (10). Furthermore, the pathogenesis of RDD is poorly understood. Studies showed that RDD were related to viral infection, including herpes viruses, Epstein–Barr virus, cytomegalovirus, and HIV (11), though their exact relationship has not been elucidated. Immune abnormalities and genetic mutations (such as KRAS and MAP2K1) may also contribute to this disease (12, 13). Thus, more studies are needed to reveal the pathogenesis, make an accurate diagnosis, and develop effective treatments.

The previous literature reported that CNS-RDD can occur in both children and adults, with a mean age of 39 years old and a male-to-female ratio of 1.8:1.0 (14). In our study, there were both child and adult patients, with an average age of 41 years old and a male-to-female ratio of 3:1, which was in accord with previous literature. Tatit et al. reviewed 287 cases of CNS-RDD and discovered that isolated intracranial RDD without extracranial lesions accounted for 77.0% (221/287), systemic RDD with CNS involvement accounted for 23.0% (66/287), CNS-RDD with isolated spinal involvement accounted for 8.0% (23/287), and CNS-RDD with spinal involvement accounted for 22.3% (64/287) (2). CNS-RDD with intraparenchymal involvement has been rarely reported (15). In our study, one spinal RDD (1/12) patient and two patients (2/12) with intraparenchymal involvement were included. The reason for the discrepancy may be the limited sample size. According to the previous literature, the cerebral convexity, parasagittal region, suprasellar region, petroclival region, and cavernous sinus were the most involved structures in intracranial RDD (15, 16). Our study revealed that cerebral convexity, petroclival region, and sellar and parasellar regions were the predilection sites, which was consistent with the previous literature.

As for imaging, most CNS-RDD represents dural-based masses resembling meningioma, which is difficult to diagnose accurately before operation (6). Other CNS-RDD with intraparenchymal involvement mimics lymphoma or tuberculous granuloma (17, 18). The typical CT imaging feature of CNS-RDD is a dura-based and homogenous mass with obvious perilesional edema and significant enhancement. Moreover, thickening or destruction of adjacent bone can also occur in some patients. On MRI, most of the CNS-RDD represents homogenous, isointense, and dura-based mass with marked homogenous enhancement (8, 19, 20). In addition to these characteristics, we found that lobulation and/or pseudopodium sign and multiple lesions in different intracranial sites were also important imaging features for some CNS-RDD. The lobulation and/or pseudopodium sign may be directly related to the involvement of the cerebral pia mater and possibly lead to postoperative recurrence (8). Multiple lesions may indicate that the lesion is aggressive and of a high risk of recurrence.

At surgery, most lesions were tough and grayish masses with rich blood supply and tightly adhered to surrounding tissues. At present, histopathological and immunohistochemical examinations are essential for the definitive diagnosis of RDD. Emperipolesis is a specific histopathological manifestation of RDD that represents the lymphocytes or erythrocytes engulfed in the cytoplasm of histiocytes (21–23). However, this phenomenon is rare in the CNS-RDD (10). Typically, immunohistochemical staining showed that these histiocytes were positive for the S-100 and CD68 proteins but negatively for the CD1a protein (16). Positive staining of the S-100 protein can help distinguish RDD from granulomatous diseases, and negative expression of CD1a protein rules out the possibility of Langerhans cell histiocytosis (10).

The treatment of CNS-RDD is still not standardized and remains controversial. Surgery, steroids, chemotherapy, and radiation therapy were all reported (24–26). Surgery was often performed when CNS-RDD was misdiagnosed as a meningioma. Surgical resection cannot only relieve compression of vital structures in the CNS but also provide enough specimens for pathological diagnosis. Previous literature showed that the extent of surgical resection was closely related to the prognosis of CNS-RDD (16, 27). In this study, seven patients achieved total resection, three patients received subtotal resection, one patient underwent partial resection, and a biopsy was performed on one patient. All these patients were closely observed after surgery. Of the seven patients who achieved total resection, only one patient (1/7) showed lesion recurrence during the follow-up, indicating that a satisfactory prognosis could be obtained through total resection of lesions, but regular postoperative follow-up was still necessary. Other treatment regimens were performed in patients with recurrence or residual lesions. Steroid therapy, as a common and noninvasiveness medical therapy, is a preferred choice for CNS-RDD (28, 29). However, some patients were not sensitive to steroid therapy. In this study, case 8 received postoperative steroid therapy, but the symptoms still progressed without effective relief. Notably, the combination of steroid therapy and chemotherapy showed promising therapeutic efficacy in progressive CNS-RDD. Case 6 was administered the combination of cytarabine, lenalidomide, and dexamethasone, which achieved a profound therapeutic effect. Moreover, case 9 was treated with the combination of prednisone, 6-mercaptopurine, methotrexate, and vinblastine, and had the disease controlled even without completing the recommended courses of treatments. Thus, chemotherapy was an effective treatment for CNS-RDD. Studies showed that radiation therapy was also effective for the local control of CNS-RDD (30, 31). However, none of the patients in our study received radiotherapy.

There were some limitations to our study. First, it was a retrospective study rather than a prospective study. The sample size is relatively small given that CNS-RDD is extremely rare. More prospective studies with larger sample sizes in multiple centers are necessary. Moreover, some latent lesions in other systems may be missed because some patients in our study did not receive an imaging examination of the whole body.

## Conclusion

Accurate diagnosis of CNS-RDD is a great challenge for clinicians. Solitary CNS-RDD is not easily distinguishable from meningioma. However, when the MRI imaging of the disease represents dura-based masses with significant edema, homogeneous enhancement, and lobulation and/or pseudopodium sign, we should consider the possibility of CNS-RDD. Surgery is an important and effective treatment for CNS-RDD. Steroids and chemotherapy in combination could play a remarkable role in the management of CNS-RDD, especially for relapsing cases or residual lesions.

## Data availability statement

The raw data supporting the conclusions of this article will be made available by the authors, without undue reservation.

## Ethics statement

This study was approved by the Ethics Committee of Xiangya Hospital of Central South University and informed consent was obtained from each patients.

## Author contributions

JY conceived and designed the study. XZ, WY, and YG wrote the manuscript. YH, ZJ, YL, BX, SZ, XJ, and QL analyzed the results. YG, and ZJ performed the image visualization. All authors contributed to the article and approved the submitted version.

## Funding

This work was supported by Natural Science Foundation of Hunan Province (No. 2021JJ41050).

## Conflict of interest

The authors declare that the research was conducted in the absence of any commercial or financial relationships that could be construed as a potential conflict of interest.

## Publisher’s note

All claims expressed in this article are solely those of the authors and do not necessarily represent those of their affiliated organizations, or those of the publisher, the editors and the reviewers. Any product that may be evaluated in this article, or claim that may be made by its manufacturer, is not guaranteed or endorsed by the publisher.

## Supplementary material

Supplementary Figure 1 Intraventricular RDD (Case 1) and brain stem RDD (Case 8). **(A-C)** Axial MRI images showed a lesion with the presence of irregular shape, peripheral edema, ambiguous brain-lesion border and moderate enhancement located in the ventricle in Case 1. **(D-F)** Axial MRI images showed multiple lesions, moderate peripheral edema, and homogenously enhanced mass located in the pons in Case 8.

Supplementary Figure 2 Chemotherapy is effective for relapsing cases. **(A)** Axial MRI image showed the lesion was recurrent (arrow) in the clivus region half a year after the operation. **(B)** An axial MRI image showed lesion disappeared completely after chemical therapy, and no recurrence was found in regular follow-ups.
