# Supplementary figures and images for: Rosai–Dorfman disease of the central nervous system: A clinical, radiological, and prognostic study of 12 cases
Source: Front Oncol. 2022 Nov 3;12:1013419. doi: 10.3389/fonc.2022.1013419 (PMC9669081; doi:10.3389/fonc.2022.1013419)

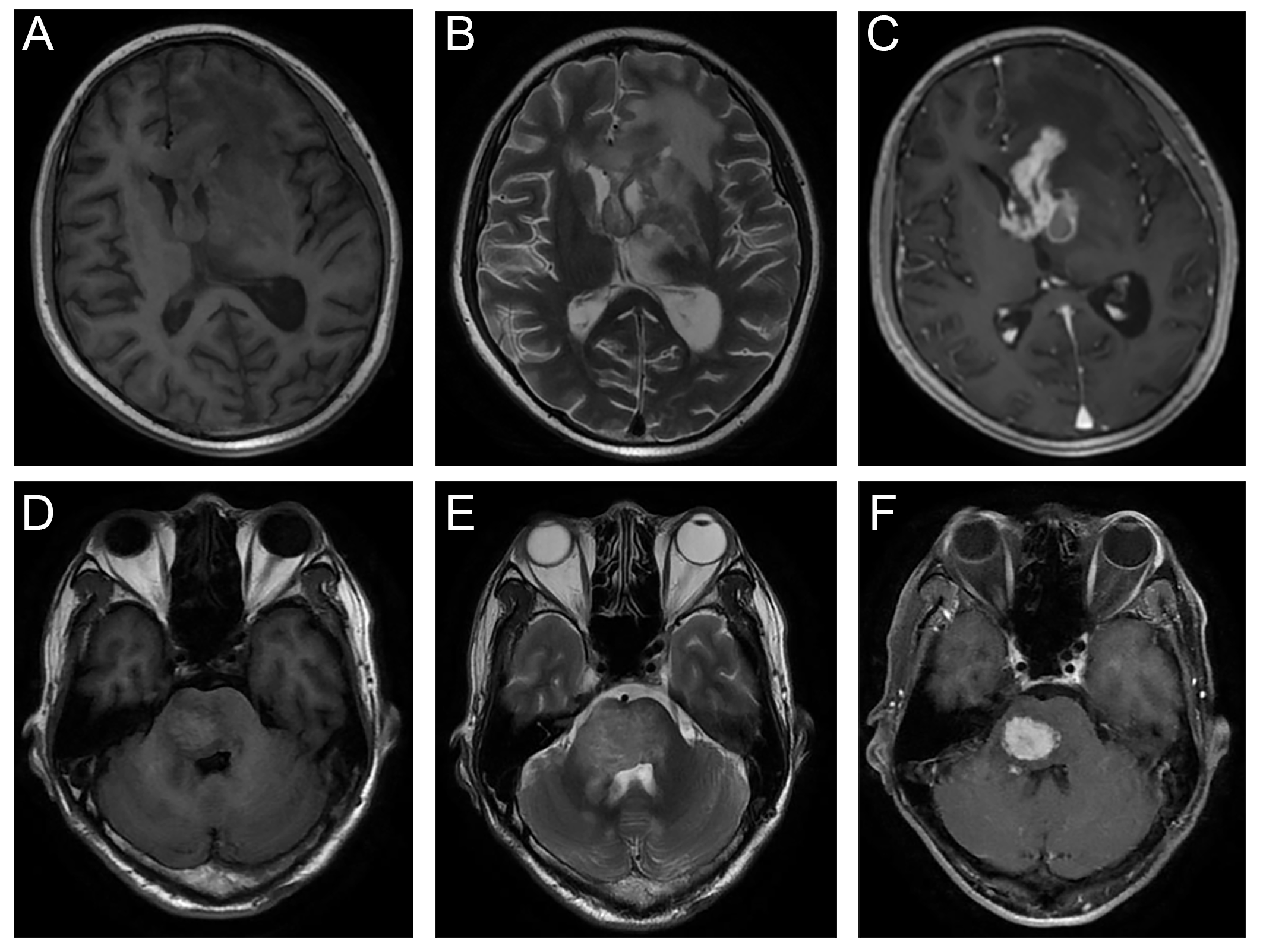

Supplement: Supplementary Figure 1 — Intraventricular RDD (Case 1) and brain stem RDD (Case 8). (A-C) Axial MRI images showed a lesion with the presence of irregular shape, peripheral edema, ambiguous brain-lesion border and moderate enhancement located in the ventricle in Case 1. (D-F) Axial MRI images showed multiple lesions, moderate peripheral edema, and homogenously enhanced mass located in the pons in Case 8. [file Image_1.tif]

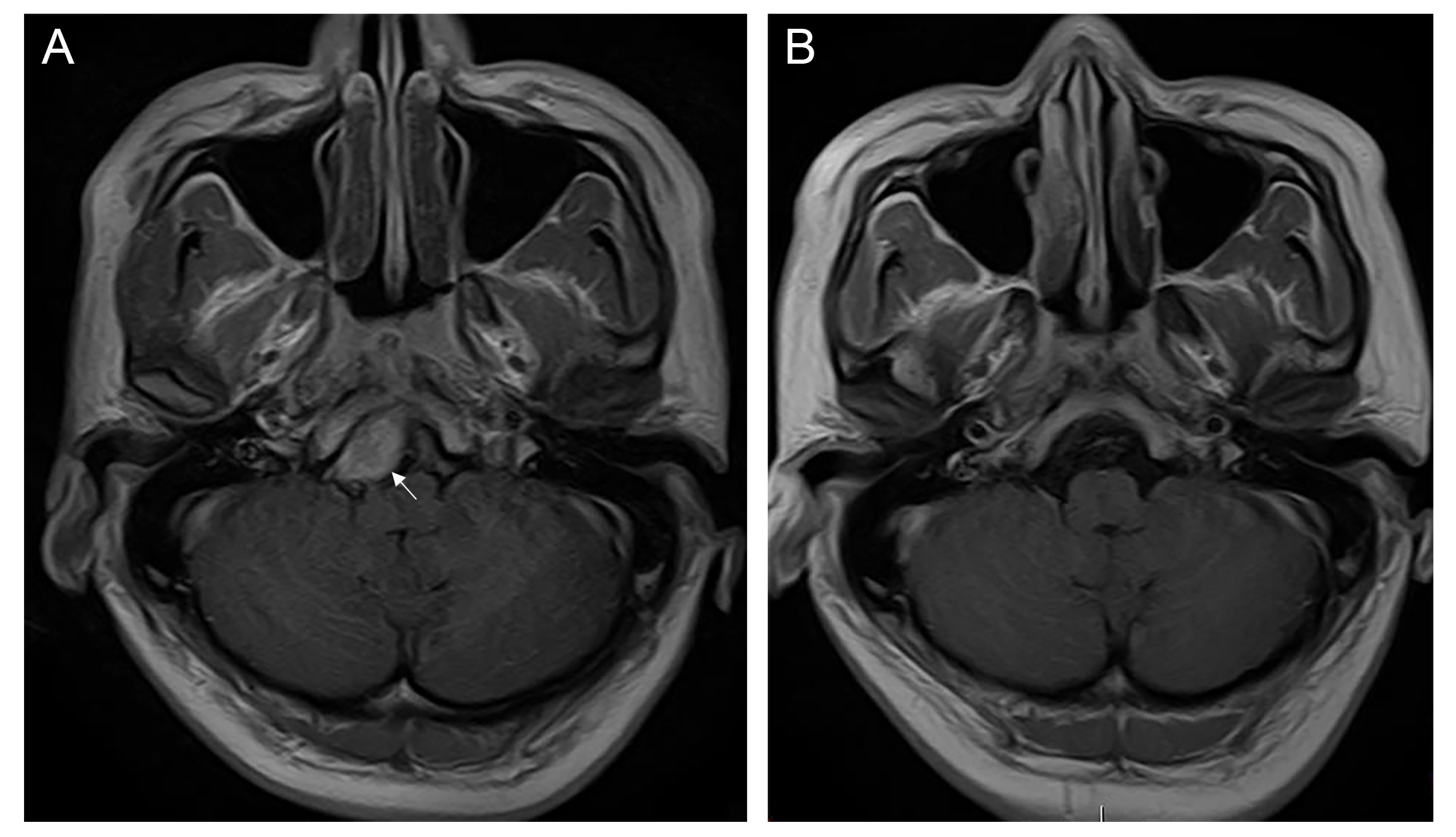

Supplement: Supplementary Figure 2 — Chemotherapy is effective for relapsing cases. (A) Axial MRI image showed the lesion was recurrent (arrow) in the clivus region half a year after the operation. (B) An axial MRI image showed lesion disappeared completely after chemical therapy, and no recurrence was found in regular follow-ups. [file Image_2.tif]
